# The reliability and quality analysis of health information about *Helicobacter pylori* on WeChat official accounts

**DOI:** 10.7717/peerj.20543

**Published:** 2026-01-27

**Authors:** Chunxi Shu, Xiaomin Zhang, Qin Zhong, Yin Zhu

**Affiliations:** 1 Department of Gastroenterology, Jiangxi Provincial Key Laboratory of Digestive Diseases, Jiangxi Clinical Research Center for Gastroenterology, Digestive Disease Hospital, Nanchang, China; 2 First Affiliated Hospital of Nanchang University, Nanchang, China; 3 Jiangxi Medical College, Nanchang University, Nanchang, Jiangxi, China

**Keywords:** *Helicobacter pylori*, WeChat official account, Health information, Quality, Reliability, Popular science article

## Abstract

**Introduction:**

* Helicobacter pylori (H. pylori)* has drawn considerable attention because of its high infection rate. Although WeChat Official accounts (WOAs) have become a prevalent source of public health information, the reliability and scientific validity of *H. pylori*-related content on the platform remain uncertain. Therefore, this study aimed to systematically evaluate the reliability and quality of health information on *H. pylori* disseminated through WOAs and propose evidence-based strategies for enhancing the standard of online health information.

**Methods:**

Articles containing the keywords “幽门螺杆菌” or “幽门螺旋杆菌” (Chinese for *H. pylori*) were retrieved from the WeChat platform. After selection, a total of 115 articles were included in this study. Subsequently, raters collectively evaluated the articles using the Journal of American Medical Association (JAMA) benchmark criteria, the modified DISCERN (mDISCERN) tool, and the Global Quality Scale (GQS). Statistical analyses were then conducted. All continuous data were described as median (interquartile range).

**Results:**

The median scores for JAMA, mDISCERN, and GQS across all articles were 2.00 (1.00), 3.00 (2.00), and 3.00 (2.00), respectively. Spearman correlation analysis revealed significant positive correlations between each pair of assessment tools (JAMA, mDISCERN, and GQS; *P* < 0.001). The Kruskal–Wallis test indicated that JAMA, mDISCERN, and GQS scores were all significantly associated with article sources (*p* < 0.001). Enterprise accounts contributed to the majority of articles (58.51%). Articles sourced from non-profit organizations demonstrated higher reliability and quality, whereas those from individual sources exhibited lower scores. The issues identified in the articles primarily concerned the treatment of *H. pylori*.

**Conclusion:**

Generally, the reliability and quality of *H. pylori* information found on WOAs was unsatisfactory. Users face a significant risk of exposure to misinformation. Content originating from non-profit organizations or large tertiary hospitals demonstrated strong correlations with higher reliability and quality scores. To address these challenges and enhance the credibility of online health information, concerted efforts are required.

## Introduction

*Helicobacter pylori* (*H. pylori*), a gram-negative bacterium infecting more than 50% of the global population (Hooi et al., 2017), is a major cause of gastrointestinal disorders, including chronic gastritis, peptic ulcers, and gastric cancer (Ding et al., 2022). *H. pylori* infection may also contribute to extra-gastrointestinal diseases, such as iron-deficiency anemia, idiopathic thrombocytopenic purpura, autoimmune diseases, and cardiovascular and cerebrovascular diseases (Santos et al., 2020). In 2014, the World Health Organization (WHO) called for the worldwide eradication of *H. pylori* as an important strategy for preventing related diseases (Elbehiry et al., 2023). Studies have shown that if patients are provided with accurate, understandable, and individualized health information, they are not only more active in detecting diseases but also more cooperative with treatment (Akbolat et al., 2023). Moreover a study has shown that misleading information will potentially aggravate the doctor-patient relationship (Wang et al., 2023). Consequently, it is necessary to investigate the quality and reliability of information about *H. pylori* that people encounter in their daily lives.

Modern technology makes it easier for people to access health information, but also brings about some risks. WeChat Official accounts (WOAs), a feature of WeChat where individuals, institutions, enterprises, and non-profit organizations can disseminate information, provide users with an enormous quantity of content each day (Shen et al., 2019). The well-defined classification of accounts enables the evaluation of information quality based on account types. Unlike short video platforms (*e.g.*, TikTok, Bilibili, YouTube), WOAs primarily feature text-based articles complemented by images, which leave a relatively deep impression on people. Therefore, misinformation on WOAs may lead to more serious consequences. Additionally, WOAs cover a broader population in China, especially among elder people and those whom rely on subscription-based push notifications, while short video platforms cater to younger audiences who depend more on algorithm-based recommendations (Feng et al., 2023). Two previous studies that assessed YouTube, TikTok, Bilibili, and Kwai as health information sources on *H. pylori* both reached the conclusion that the information quality was not satisfactory and few practical recommendations were put forward (Lai et al., 2023; Ergenç & Uprak, 2023).

Therefore, this study was designed to better understand the reliability of *H. pylori*-related information on WOAs and propose actionable improvement measures and recommendations. Articles published on WOAs concerning *H. pylori* were gathered and assessed by three commonly employed standard scales. Factors affecting quality were analyzed with the aim of improving online health information and helping patients find reliable advice.

## Materials & Methods

### Search strategy and article collection

**Figure 1:**
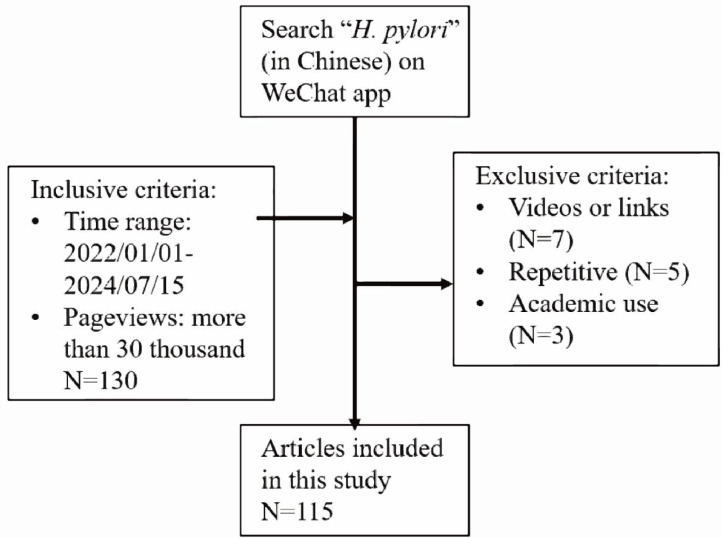
Selection process. Article selection on WeChat Official accounts (WOAs).

Articles were initially retrieved from the WeChat platform using the keywords “幽门螺杆菌” or “幽门螺旋杆菌” (*H. pylori* in Chinese), and the search results were sorted by descending view count. To balance recency and public engagement, articles published between December 31st, 2021 and July 15th, 2024 with over 30,000 pageviews were selected. Exclusion criteria were then applied: (1) articles consisting solely of videos or hyperlinks; (2) duplicate content; and (3) materials restricted to academic purposes. After this screening process, 115 articles met the inclusion criteria. The selection process is detailed in Fig. 1.

### Classification of article sources and contents

Building upon prior research and current search results, the articles were classified into four groups based on publisher type: (1) individuals (*e.g.*, physicians), (2) enterprises (*e.g.*, companies), (3) institutions (*e.g.*, National Health Commission), and (4) non-profit organizations (*e.g.*, academic associations). The content was categorized into key domains related to *H. pylori* infection, symptoms, examination, treatment, prevention, and recrudescence. Notably, most articles addressed multiple topics within these categories.

### Evaluation tools

The Journal of American Medical Association (JAMA) benchmark criteria (Table 1) were proposed by Silberg, Lundberg & Musacchio (1997) in order to assess the reliability of medical information on the Internet. Each of the four dimensions is awarded with one point. Each domain is assigned one point if the article meets predefined standards, resulting in a maximum score of 4 (highest reliability) and a minimum score of 0 (lowest reliability) (Silberg, Lundberg & Musacchio, 1997). Additionally, the modified DISCERN (mDISCERN) criteria were adopted to assess the articles from another perspective (Table 2). DISCERN was initially created to judge the quality of written consumer health information on treatment choices (Charnock et al., 1999). It was then was modified to systematically evaluate the completeness of diverse health information (Singh, Singh & Singh, 2012). This tool includes five yes/no questions, with each affirmative response earning one point (total range: 0–5). Higher scores reflect greater content comprehensiveness. Finally, to measure the quality and flow of the articles, we applied the Global Quality Scale (GQS) criteria (Table 3), a five-grade scale where the higher the grade, the better the quality (Bernard et al., 2007).

**Table 1:** Journal of American Medical Association (JAMA) benchmark criteria.

**Criterion**	**Description**
Authorship	Authors and contributors, their affiliations, and relevant credentials should be provided
Attribution	References and sources for all content should be listed clearly, and all relevant copyright information noted
Disclosure	“Ownership”, sponsorship, advertising, underwriting, commercial funding arrangements or support, or potential conflicts of interest should be prominently and fully disclosed
Currency	Dates that content was posted and updated should be indicated

**Table 2:** Modified DISCERN (mDISCERN) criteria.

**Item**	**Description**
1	Are the aims clear and achieved?
2	Are reliable sources of information used? (*i.e.*, publication cited; provided by certified orthopedists or neurosurgeons)
3	Is the information presented balanced and unbiased?
4	Are additional sources of information listed for patient reference?
5	Are areas of uncertainty mentioned?

**Table 3:** Global quality scale (GQS) criteria.

**Description**	**Score**
Poor quality and flow, most information missing; technique misleading; unlikely to be useful for patient education	1
Generally sparse quality and flow, some information provided but many important topics missing; technique poor; of very limited use to patients	2
Moderate quality and suboptimal flow, some important information provided adequately but others poorly discussed; technique basically adequate; somewhat useful for patients	3
Good quality and generally good flow, majority of information provided but some topics not covered; technique almost adequate; useful for patients	4
Excellent quality and flow, full information provided; technique adequate; highly useful for patients	5

### Rating process

To be more scientific, two raters (an expert in gastroenterology, Yin Zhu, and a medical student well-versed in current *H. pylori* management guidelines and relevant clinical knowledge, Xiaomin Zhang) first separately rated 10 articles selected randomly from the 115 articles using the criteria. The points of disagreement were then discussed to ensure the two raters had a consistent understanding of each criterion during formal scoring. After the controversial items discussed, the two raters jointly evaluated each article through real-time discussion until a consensus score was reached.

### Statistical analysis

For the data analysis, we utilized IBM SPSS Statistics 27 (IBM Corp., Armonk, NY, USA) and Microsoft Office Excel 2016 (Microsoft, Redmond, WA, USA). Descriptive statistics were calculated to assess the reliability, accuracy, and characteristics of the articles, which included mean, median, interquartile range, and minimum and maximum values. The Kruskal–Wallis test, a nonparametric statistical test, was executed to evaluate differences in variables based on different article sources between groups. Subsequently, Spearman correlation analysis was performed to assess the correlations between the JAMA, mDISCERN, and GQS scores. All reported *P* values were two-sided, and *P*-values <0.05 indicated a statistically significant difference.

## Results

### Sources and contents of included articles

**Figure 2:**
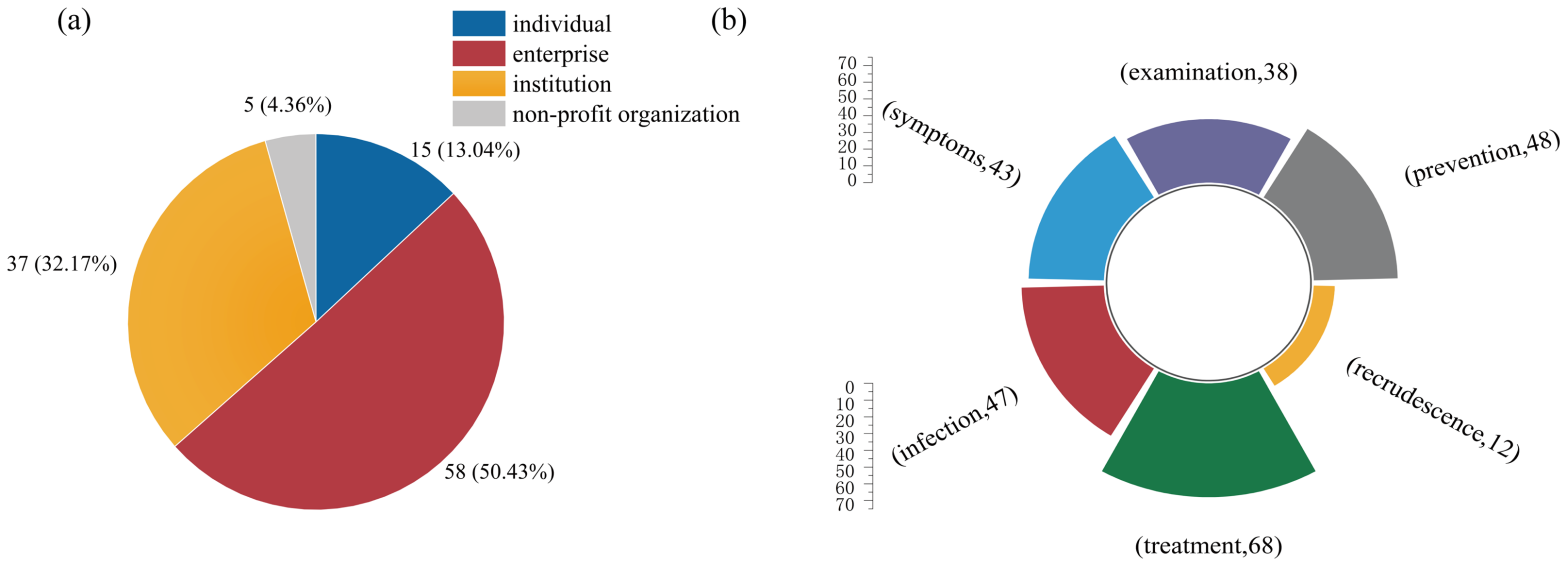
(A) Article sources. (B) Article content. (A) More than half of the articles came from enterprise accounts. (B) Only 12 articles mentioned recrudescence.

A total of 115 articles were included in this study. Articles from enterprises accounted for the largest proportion (58.51%), followed by institutions (37.32%), individuals (15.13%), and non-profit organizations (5.4%) (Fig. 2A). Regarding content focus, the most frequently addressed topic related to *H. pylori* was treatment, followed by infection, symptoms, and examination, while only 12 articles (10.43%) mentioned recrudescence (Fig. 2B).

**Figure 3:**
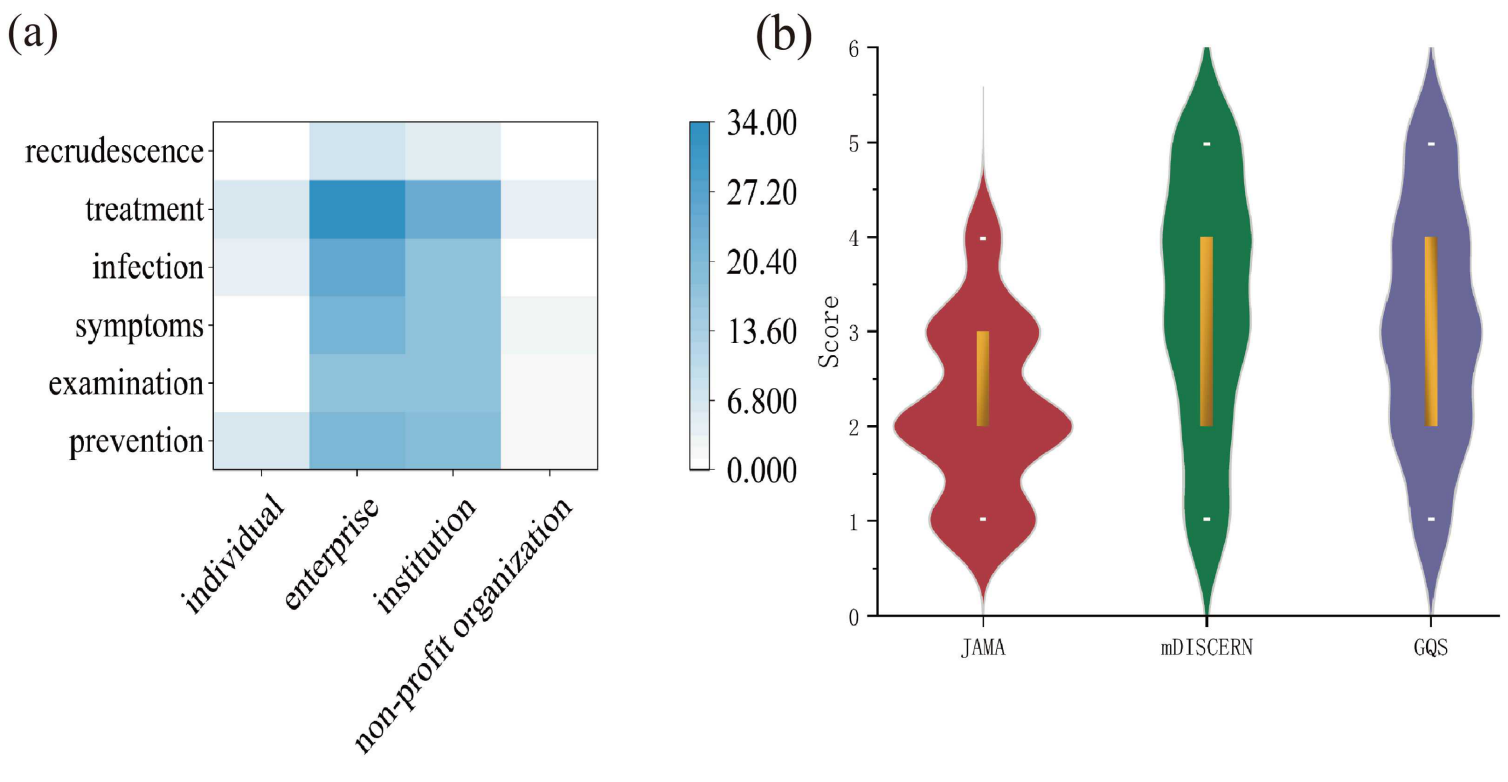
(A) Heatmap of article counts; (B) Violin plot of scores.

The heatmap (Fig. 3A) provides a general overview of article distribution across sources and content categories. Regardless of source, treatment was the most frequently addressed topic. Enterprise and institutional accounts demonstrated the broadest thematic coverage, while articles from individuals and non-profit organizations covered only three to four topics, with neither group mentioning recrudescence.

### Evaluation of articles

The average scores for JAMA, mDISCERN, and GQS were 2.19, 3.26, and 3.05, respectively, with median values of 2.00 (1.00), 3.00 (2.00), and 3.00 (2.00). The violin plot (Fig. 3B) visually summarizes the score distributions. Detailed scores are presented in Table 4. Spearman correlation analysis (Table 5) revealed significant positive correlations between each pair of JAMA, mDISCERN, and GQS scores (*P* < 0.001).

**Table 4:** Evaluation results of the articles.

		**N**	**Average**	**Median (IQR)**
JAMA	1 score	28	2.19	2.00 (1.00)
	2 score	47		
	3 score	30		
	4 score	10		
mDISCERN	1 score	16	3.26	3.00 (2.00)
	2 score	16		
	3 score	29		
	4 score	30		
	5 score	24		
GQS	1 score	13	3.05	3.00 (2.00)
	2 score	27		
	3 score	33		
	4 score	25		
	5 score	17		

**Notes.**

IQR, interquartile range.

**Table 5:** Spearman correlation analysis between JAMA, mDISCERN, and GQS.

		**JAMA**	**mDISCERN**	**GQS**
JAMA	CC	1.000	0.590	0.570
	P	–	<0.001	<0.001
mDISCERN	CC	0.590	1.000	0.772
	P	<0.001	–	<0.001
GQS	CC	0.570	0.772	1.000
	P	<0.001	<0.001	–

**Notes.**

CC, correlation coefficient.

### Quality of articles with different contents from different sources

**Figure 4:**
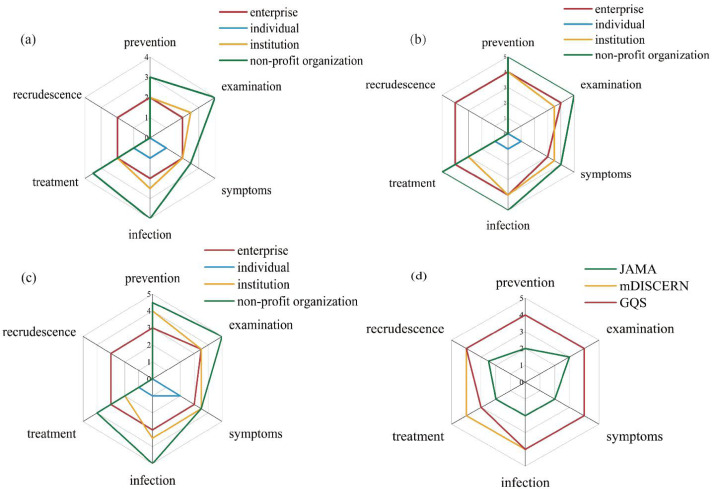
Comparison of content across different scores. (A) JAMA score; (B) mDISCERN score; (C)GQS score; (D) overall comparison of different content types by JAMA, mDISCERN and GQS scores.

Table 6 shows significant associations between article sources and JAMA, mDISCERN, and GQS scores (*p* < 0.001). Articles from non-profit organizations exhibited higher scores across all three metrics, whereas articles from individual accounts showed lower scores. Regarding the relationship between content and scores, Fig. 4 illustrated that non-profit organizations provided higher-quality information on examination and infection. Institutional articles covering examination and infection achieved superior JAMA scores, while those covering prevention and infection ranked higher in mDISCERN. Prevention-focused institutional articles attained the highest GQS scores. As for articles from individual accounts, they not only addressed fewer topics but also demonstrated lower overall quality. Overall, as shown in Fig. 4D, examination-focused articles achieved the highest median JAMA scores, whereas treatment-related content ranked lowest in GQS compared to other topics.

**Table 6:** Score analysis of articles from different sources.

		**median (IQR)**	**H**	***P* value**
JAMA	Enterprise	2.0 (1.0)	20.637	<0.001
	Individual	1.0 (1.0)		
	Institution	2.0 (1.0)		
	Non-profit organization	2.5 (2.5)		
mDISCERN	Enterprise	4.0 (1.0)	28.132	<0.001
	Individual	1.0 (0.0)		
	Institution	4.0 (2.0)		
	Non-profit organization	5.0 (1.5)		
GQS	Enterprise	3.0 (1.0)	21.584	<0.001
	Individual	1.0 (1.0)		
	Institution	4.0 (2.0)		
	Non-profit organization	4.5 (2.5)		

**Notes.**

IQR, interquartile range.

## Discussion

According to the guidelines from national health education professional organizations, public health education is a major part of the public health service system (Sun et al., 2021). In China, WOAs rank among the most widely-used public health education channels due to their broad reach and impact (Zhang et al., 2017), though their application in delivering health-related information remains in its nascent stage. To our knowledge, this is the first study evaluating the quality of *H. pylori*-related health information on WOAs.

### Enterprises: the largest information source

Tencent categorizes WOAs into four types: individual, enterprise, institutional, and non-profit organization. Readers can identify account categories through profile descriptions, which enhances transparency in information disclosure. Enterprise accounts refer to those generating profit through services (*e.g.*, online consultation) and aligning with commercial accounts in other search engines, such as Baidu (Li et al., 2021). In this study, individual accounts primarily belonged to traditional Chinese medicine practitioners while enterprise accounts were operated by companies engaged in pharmaceutical sales and providing online consultation services. Public hospitals or the Centers for Disease Control and Prevention ran institutional accounts. Prior research, such as an evaluation of online cauda equina syndrome information quality (O’Neill et al., 2014), found most health websites to be academically or physician-curated. In a study that analyzed the reliability and educational quality of YouTube videos as a source of information on pediatric scoliosis (Rudisill et al., 2022), the majority of videos were posted by physicians. In contrast, this study found enterprise accounts to be the most prevalent (51%), aligning with findings from an earlier study (Yang et al., 2022). This may stem from the antibiotic-based triple or quadruple therapy for *H. pylori* treatment, which makes it possible for enterprises to promote medication sales and explains their focus on treatment content (Liou et al., 2020). However, due to the antibiotic resistance of *H. pylori* and patients’ health status, it is suggested that patients seek professional help before medication use (Du et al., 2020). Moreover, some for-profit organizations (for example, enterprises) exploit social media features to exaggerate facts, which fosters misinformation among users (Nian & Sundararajan, 2022). Hence, enterprise-sourced content risks may be misleading and potentially hinder eradication efforts. It is worth noting that most articles from enterprise accounts emphasized family-based *H. pylori* testing and treatment, which raised public awareness and helped prevent transmission at family level—a strategy that may ultimately aid *H. pylori* eradication.

### Quality of *H. pylori* related health information on WOA

The current results indicate that the reliability and quality of *H. pylori*-related health information on WOAs are unsatisfactory, with median JAMA, mDISCERN, and GQS scores of 2.00 (1.00), 3.00 (2.00) and 3.00 (2.00), respectively. This suggests that casual internet users or patients seeking health information online are exposed to a high risk of being misled, which will likely shape their perspectives on this disease in a way that is at odds with professional medical advice, reduce patients’ compliance, and further aggravate doctor-patient conflicts (Liu, Zhang & Lu, 2019).

In prior studies, Yang et al. (2022) investigated hypertension treatment–related information on WOAs and confirmed its low quality. Similarly, Pan et al. (2024) assessed cancer-related WeChat public accounts and found insufficient credibility. In contrast, Ng, Lim & Fong (2020) reported satisfactory quality in English-language YouTube videos on systemic lupus erythematosus and noted their reliability and educational value. The differences in these conclusions may be attributed to the discrepancy of the studied fields and platforms.

The poor quality and reliability of articles about *H. pylori* on WOAs might be related to the absence of robust regulation and censorship, which enables unqualified accounts to publish unprofessional and unsubstantiated information. Notably, some articles were titled with phrases such as “dietary therapy” which may cater to patients’ psychological needs. These articles conveyed subjective messages with bias or even wrong information which might cause anxiety or negligence among the public. For instance, in an article called “Eat raw garlic to prevent *H. pylori* infection”, the author claimed that consuming three to five raw garlic cloves can prevent *H. pylori* infection without evidence. However, it has been proven that garlic intake for long durations did not appear to have an effect on the prevalence of *H. pylori* infection (Salih & Abasiyanik, 2003).

Among the analyzed articles, few comprehensively covered all six key concerns regarding *H. pylori* (prevention, infection, symptoms, examination, treatment and recrudescence) and the contents were quite homogeneous. For instance, while most articles correctly identified the ^1^^3^C/^1^^4^C urea breath test as the primary non-invasive diagnostic method, only 12% mentioned family-based screening recommendations —-a critical omission given China’s 71.21% familial infection rate (Zhou et al., 2023). In terms of prevention, using public chopsticks, reducing dining out, and avoiding leftovers were correctly emphasized. Analysis revealed content inaccuracies and biases were predominantly in treatment-related discussions. Although global guidelines recommend a 10- or 14-day course of bismuth-containing quadruple therapy for, numerous individual accounts promoting traditional Chinese medicine disregarded this consensus. These accounts advocated for the use of Chinese herbal medicine without certification and evidence. These articles typically lack author/contributor credentials, citations, source references, or proper content copyright information, and offer limited clinical value for patients, characteristics that collectively explain their consistently poor JAMA, mDISCERN, and GQS scores. This misleading information may not only lead to failure in eradicating *H. pylori* but also compromise the efforts of subsequent eradication treatment. Despite the generally unsatisfactory quality of the articles, exceptions existed. For example, articles from the non-profit organization China Popular Science and some major general hospitals exhibited complete coverage, factual accuracy, and credible sourcing. Our analysis revealed a strong correlation between article quality and reliability. The higher the reliability, the better the quality. Although articles from non-profit organizations demonstrated the highest quality, their limited quantity was attributed to the scarcity of accounts operated by such entities. The shortcomings of articles published by general hospitals were also evident. Essentially, information from professional medical workers is considered trustworthy, but these articles often lacked author credentials and references, substantially undermining their reliability for patients.

### Future directions

Health education influences all aspects of one’s daily and social life, including eating, drinking, entertaining, working, and learning. The general public absorbs health information from social media more frequently than ever with the development of handheld mobile devices. However, misleading health information may complicate disease self-management. Consequently, it is crucial to improve the quality of articles published on WOAs, given WeChat is one of the most commonly-used social media platforms. To this end, we propose the following suggestions to make WOAs a truly credible tool for health education among the public. First, to effectively combat health misinformation, regulatory agencies and platforms should work together to establish robust monitoring systems for health-related content on WOAs in order to minimize the spread of false or misleading claims. Platforms can take additional steps, such as automatically attaching links to official sources or fact-checking websites when health-related articles are uploaded, to help readers verify information (Barman & Colan, 2023). AI-generated warning labels could also flag potentially unreliable content, and prompt authors to double-check facts and caution readers against blind trust (Kairam, Mercado & Sumner, 2022). At the same time, all accounts should be prohibited from posting unverified health claims but instead provide clear references, which not only helps patients access accurate information but also improves the credibility and reach of their content. Academics and medical professionals play a key role as well, as they could contribute more to public health education by creating clear, evidence-based articles. Public awareness is equally important: integrating media literacy into school curricula could teach people to critically evaluate online health information, which in turn would pressure content creators to improve accuracy (Jafar et al., 2023). For topics like *H. pylori*, articles should cover key patient concerns—such as treatment side effects and whether all positive cases require eradication—while authoritative institutions could collect public questions and host expert-led Q&A sessions to improve health literacy.

### Limitations

It is necessary to acknowledge that our study had some limitations. First, although two researchers participated in the rating process, unavoidable subjective factors influenced scoring outcomes. Second, at the article collecting stage, the search results were displayed by descending viewpoint count. However, below the threshold of 30,000 pageviews, the number of results dropped sharply, and the sorting became irregular. Therefore, to balance between methodological rigor and capturing articles with meaningful public impact, the threshold was set at 30,000 pageviews for this study. Consequently, articles with relatively low pageviews were inevitably excluded, regardless of quality. Third, beyond the standards we used to evaluate the quality and reliability of articles, additional factors such as webpage aesthetics, user-friendliness, and customized and individualized content remained unmeasured by the three tools. Furthermore, while focusing on content quality, readability and clarity were overlooked. This suggests potential dimensions for future research.

## Conclusion

WOAs have shown great potential in spreading public health education, yet the quality of this information varies. This study reveals that the overall quality of *H. pylori*-related articles published on WOAs was unsatisfactory. There is a high likelihood that users searching for information about *H. pylori* on WOAs encounter inaccurate, incomplete, or even misleading information. Optimizing the overall quality and reliability of articles on WOAs requires collaborative efforts from multiple parties. The platform needs stricter admission criteria and a more comprehensive regulatory system. We recommend that health-related articles include clearly listed references and sources. The public should be encouraged to distinguish reliable information from non-credible content.

##  Supplemental Information

10.7717/peerj.20543/supp-1 Supplemental Information 1 Raw data

## List of abbreviations

WOAs: WeChat Official accounts
*H. pylori*: *Helicobacter pylori*
JAMA: Journal of American Medical Association
mDISCERN: modified DISCERN
GQS: Global Quality Scale

## Additional Information and Declarations

### Data Availability

The following information was supplied regarding data availability: The raw data is available in the Supplemental Files.
